# Outcomes and complications of distal humeral hemiarthroplasty for distal humeral fractures – A systematic review

**DOI:** 10.1177/17585732211023100

**Published:** 2021-06-17

**Authors:** Ann M Wilfred, Shakib Akhter, Nolan S Horner, Ahmed Aljedani, Moin Khan, Bashar Alolabi

**Affiliations:** 1 Faculty of Health Sciences, McMaster University, Hamilton, ON, Canada; 2 Department of Health Research Methods, Evidence, and Impact, McMaster University, Hamilton, ON, Canada; 3 Division of Orthopaedic Surgery, McMaster University, Hamilton, ON, Canada

**Keywords:** Distal humeral hemiarthroplasty, total elbow arthroplasty, systematic review

## Abstract

**Background:**

Distal humeral hemiarthroplasty has been performed for a variety of indications with the most common being management of distal humeral fractures. This systematic review evaluates the outcomes and complications of distal humeral hemiarthroplasty for this pathology.

**Methods:**

We searched PubMed, EMBASE, and MEDLINE for studies reporting indications and outcomes of patients undergoing distal humeral hemiarthroplasty. Study screening, risk of bias assessment, and data extraction were performed. Summery statistics were provided.

**Results:**

We included 11 studies (*N* = 163) in this review. In all studies, the indication for distal humeral hemiarthroplasty was the presence of an intraarticular, comminuted, unreconstructable fracture. The mean post-operative MEPS, FullDASH, and QuickDASH (SD) scores were 83.6 (6.1) points, 25.4 (10.3), and 15.7 (7.4) points, respectively. The mean post-operative range of motion (SD) was 106° (11°) in the flexion and extension arc and 153° (19°) in the protonation and supination arc. The overall rate of adverse events and complication was 63%. The rate for major complications was 11%. The mean total revision rate was 4% (0% to 15) and total re-operation rate was 29% (0% to 88%).

**Conclusion:**

Distal humeral hemiarthroplasty is a suitable option for unreconstructable distal humeral fractures and offers good functional outcomes with acceptable complication rates.

## Introduction

Adult distal humeral fractures comprise between 2% and 5% of total fracture prevalence and roughly 30% of all reported elbow fractures.^1^ Commonly employed interventions in the management of distal humeral fractures include open reduction internal fixation (ORIF) and total elbow arthroplasty (TEA).^2^ Although ORIF is considered the gold standard treatment of distal humerus fractures, it can present challenges in severely comminuted fracture patterns, where a large variability in ORIF outcomes is observed.^1,3^ TEA may be indicated when satisfactory elbow reconstruction is unachievable due to osteoporosis and/or comminution.^4^ Conservative methods of treating a fracture of the distal humerus, including the ‘bag of bones’ technique in which the position of the displaced fragments is accepted and early movement encouraged, are now rarely considered as they are thought to give poor functional results.^5^

A less commonly used alternative for treatment of unreconstructable distal humeral fractures is distal humeral hemiarthroplasty (DHH). DHH involves replacement of the distal humerus by a humeral component of a convertible total elbow system mounted with an anatomical spool.^6^ A previously published review reported the complication rate of DHH being comparable to ORIF or TEA, although the follow-up data for DHH are shorter when compared to TEA.^7^ The complication profile after DHH is different from TEA. With DHH, there is no polyethylene wear, peri-prosthetic fracture or loosening around the ulnar component or un-coupling of the linkage, which is a common reason for TEA failures.^1,6–9^ Additionally, DHH eliminates polyethylene particulate debris,^2^ and may be more suitable for active patients or those intolerable to weight lifting restrictions required in TEA.^4,10,11^ This study aims to systematically review the literature and report the indications, outcomes and adverse events (AE), and complications of DHH.

## Methods

### Literature search

We searched PubMed, MEDLINE, and EMBASE from inception to 14 April 2020. We also searched grey literature and screened registries including clinicaltrials.gov, World Health Organization (WHO), International Clinical Trials Registry Platform (ICTRP), and the International Clinical Trials Registry Platform (ISRCTN) for completed or ongoing but unpublished studies (supplementary Appendix Table 1). No language restrictions were applied. Experts within the field were contacted to see if they are aware of other relevant studies.

### Inclusion and exclusion criteria

We performed title and abstract screening independently and in duplicate using the Covidence online software. If a reviewer deemed a study relevant, it was retrieved for full-text review. We resolved disagreements in eligibility through discussion. Agreement of reviewers’ assessment for study eligibility was calculated using Cohen’s kappa coefficient (κ), with κ ≥ 0.65 being considered adequate. Eligible studies met the following criteria: (1) Included adults (≥18 years of age) undergoing DHH for distal humeral fractures; (2) Had a minimum follow-up of 12 months; and (3) Randomized trials and observational studies. We excluded individual case reports, reviews, biomechanical studies, and cadaveric studies.

### Data extraction

Two reviewers independently extracted data from each study using a Microsoft Excel data form. From each included study, the following information was extracted: publication year, last name of first author, country or countries in which study is conducted, total number of cases, mean age in years and range, the male/female ratio, and mean follow-up time in months.

### Risk of bias and quality assessment

The revised and validated version of the MINORS risk of bias tool was used to assess for any risk of bias of the included studies.^12^ A study is granted a score of 0, 1, or 2 points for not reporting, inadequately reporting, or adequately reporting information regarding a given methodological item, respectively. Eight methodological items are used for non-randomized studies for a maximum obtainable score of 16 points, while 4 more additional methodological items are used for evaluating comparative studies, which leads to a maximum obtainable score of 24 points.^12^ Reviewers assessed the risk of bias independently and in duplicate. Disagreements were resolved through discussion between the two reviewers.

### Revision and re-operation rates

Often in the literature, the terms “re-operation” and “revision” are used interchangeably. For the purposes of our review, we defined a “revision” as solely a revision arthroplasty. We defined re-operation as any subsequent surgical procedure occurring after the original elbow hemiarthroplasty procedure, which may encompass revision arthroplasty.

The revision rate for each study was calculated by dividing the number of revisions by the total number of patients in the study and multiplying by 100. The mean revision rate was calculated by taking the average of the revision rates of all included studies. An individual study’s re-operation rate as well as the mean re-operation rate was calculated in an exactly analogous manner.

## Results

### Literature search and reviewer agreement

**Figure 1.**
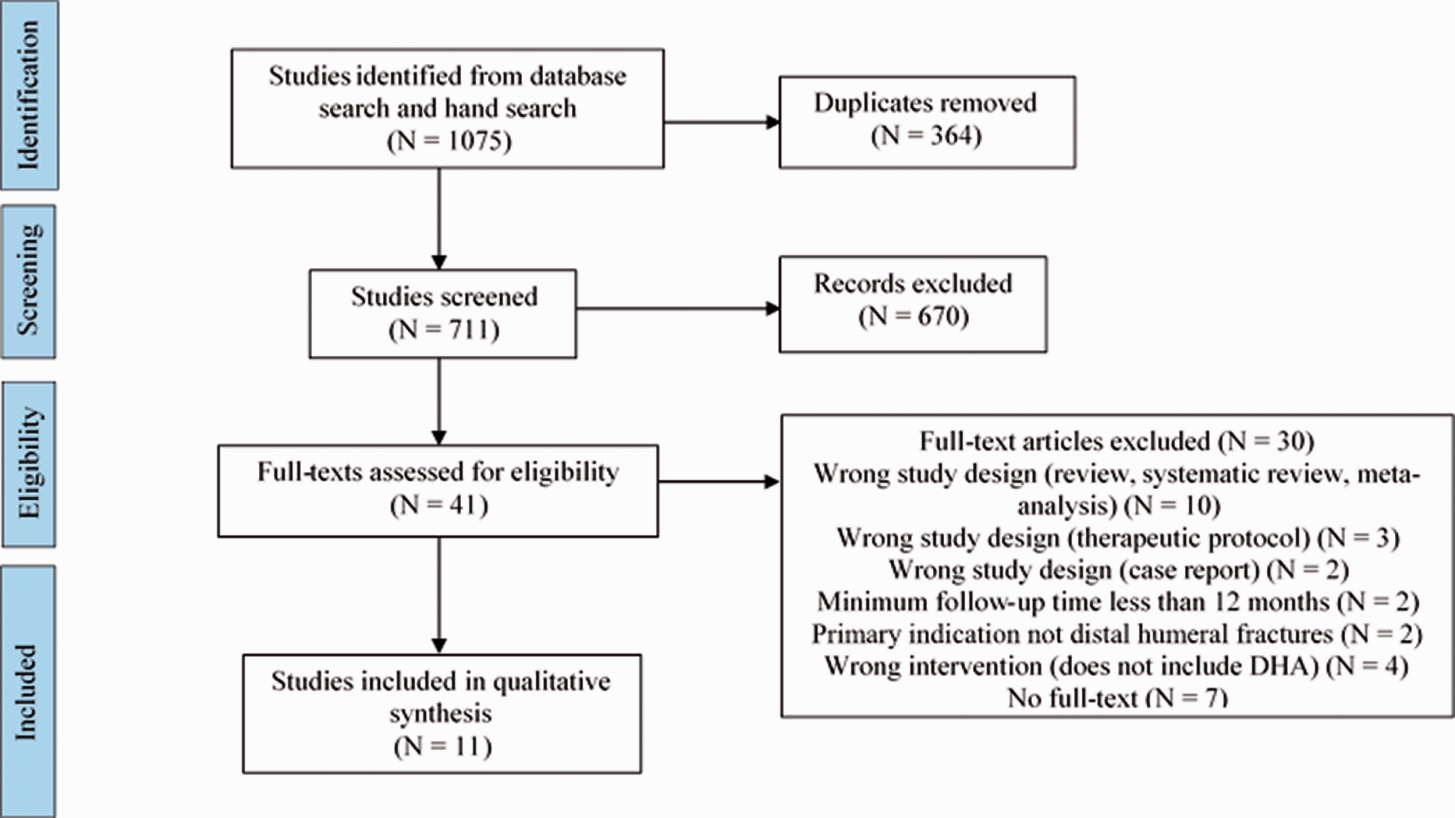
PRISMA systematic review flow diagram of study selection.

We identified 711 studies for title and abstract screening. Of these, 41 were reviewed in full text. A total of 11 observational studies were included in the qualitative synthesis (Figure 1). None of the included studies had control or comparative groups. No additional trials were identified from the gray literature. Agreement between the reviewers for study eligibility was moderately high (κ = 0.85, 95% CI: [0.78, 0.91], *P* < 0.0001).

### Study characteristics

Eleven studies (*N* = 163 patients) were included in the systematic review (Table 1).^1,3,4,6,11,13–18^ The publication date ranged from 2005 to 2019. The mean age (SD) was 67 (10) years, with patients ranging from 29 years to 90 years of age. The mean follow-up (SD) of included studies was 49 (23) months and ranged from 12 months to 81 months. There were nine retrospective case series,^3,4,6,11,13,15–18^ one retrospective cohort study,^1^ and one prospective cohort study^14^ both of which were non-comparative. With respect to the prosthesis used, one study used a Kudo humeral implant,^13^ seven studies used a Latitude implant,^1,3,4,6,14–16^ and three studies used a Sorbie-Questor implant.^11,17,18^ Five studies were conducted in Europe, two in Sweden,^13,14^ one in Denmark,^1^ one in Netherlands,^5^ and one in U.K.^15^ Three studies were conducted in North America (all in USA^3,4,16^), two studies were conducted in Australia,^17,18^ and one study was conducted in both the USA and Australia.^11^

**Table 1.** Patient demographics of included studies.

Year	First author	Level of evidence	Country	No. of cases	Mean age (range)	Male/ female	Prothesis method	Mean follow-up time in months (range)
2012	Adolfsson	IV	Sweden	8	79 (71–89)	0/8	Kudo humeral implant	54 (30–72)
2019	Al-Hamdani	IV	Denmark	24	65 (47–80)	3/21	Latitude implant	25 (12–70)
2012	Argintar	IV	USA	10	73.40 (56–77)	1/9	Latitude implant	12
2015	Heijink	IV	Netherlands	6	69 (55–77)	0/6	Latitude implant	54 (21–76)
2014	Hohman	IV	USA	7	64 (33–75)	2/5	Latitude implant	36 (26–60)
2015	Nestorson	IV	Sweden	42	72 (56–84)	3/39	Latitude implant	34.3 (24–75)
2005	Parsons	IV	USA, Australia	8	61 (46–83)	NR	Sorbie-Questor implant	NR
2015	Phadnis	IV	UK	16	78.7 (60–90)	3/13	Latitude implant	35 (24–75)
2017	Schultzel	IV	USA	10	71.9 (56–81)	NR	Latitude implant	73.2 (36–96)
2013	Smith	IV	Australia	26	61.2 (29–92)	3/23	Sorbie-Questor implant	80 (25–133)
2016	Smith	IV	Australia	6	45.1 (29–52)	2/4	Sorbie-Questor implant	81 (24–133)

### Risk of bias assessment

The risk of bias assessment using the MINORS tool revealed several key patterns in the included studies (supplementary Appendix Table 2). Out of a maximum of 16 points, the overall quality (SD) was poor with a mean score of 8 (3) points. Generally, almost all studies had a follow-up period appropriate to study aim, and most studies had a clearly stated aim and proper inclusion of consecutive patients. None of the included studies calculated a prospective sample size, most studies did not have an unbiased assessment of the study endpoint or prospective collection of data.

### Indications for DHH

For three of the included studies, the indication for DHH was solely the presence of an acute, non-reconstructable, comminuted, intraarticular distal fracture, where DHH was the primary, index procedure performed. In the remaining eight studies, DHH was also performed to treat a non-reconstructable fracture or for salvage of a previous failed fixation, nonunion or malunion after operative or non-operative treatment. In total, 145 patients from 11 studies underwent DHH as the primary index operation to treat acute distal humeral fractures,^1,3,4,6,11,13–18^ and 18 patients from 6 studies underwent DHH after previous failed fixation.^3,6,11,13,17,18^ Seven studies reported the surgical technique. Out of the total 81 patients from these 7 studies, 60 patients underwent olecranon osteotomy, 20 patients underwent a triceps-sparing approach, and 1 patient underwent a medial epicondyle osteotomy. Three studies (*N* = 39) reported additional details performed as part of the DHH procedure; Hohman et al.^3^ reported lateral column plating done on 2 patients; Smith et al.^17^ reported the fixation of 17 columnar/epicondyle fractures and 17 epicondyle fractures; and similarly, Smith et al.^18^ treated 2 columnar/epicondyle fractures, and 5 epicondyle fractures, with plate fixation.

### Outcomes

#### MEPS

The MEPS score was reported in 10 of the 11 included studies (*N* = 155 patients). ^1,3,4,6,13–18^ Of these, two studies (*N* = 20) reported only the mean MEPS score,^4,16^ two studies (*N* = 12) mentioned only the number of patients with excellent, good, fair, and poor grading,^6,18^ and six (*N* = 123) studies reported both.^1,3,13–15,17^ Out of a maximum of 100 points, the mean post-operative MEPS (SD) was 83.6 (6.1), with a range of 50 to 100 (Table 2).

**Table 2.** Outcome data from included studies.

Year	First author	Sample size	DASH score	MEPS score	ROM F/E Arc	ROM P/S Arc	Revision rate
2012	Adolfsson	8	NR	90.6 ± 5.0	NR	NR	1 (12.5%)
2019	Al-Hamdani	24	NR	82.9 ± 13.1	106 ± 22	153 ± 14	0 (0.00%)
2012	Argintar	10	35.1 ± 24.0	77.0 ± 16.0	102 ± 25	133 ± 15	0 (0.00%)
2015	Heijink	6	18.5 ± 22.0	78.33 ± 25.4	96 ± 17	165 ± 14	NR
2014	Hohman	7	33.4 ± 25.0	72.43 ± 18.0	96 ± 22	161 ± 14	0 (0.00%)
2015	Nestorson	42	20.3 ± 17.0	90.0 ± 14.4	105 ± 22	178 ± 5	NR
2005	Parsons	8	NR	NR	NR	NR	NR
2015	Phadnis	16	11.2 ± 5.8^a^	89.6 ± 5.0	116 ± 14	172 ± 9	0 (0.00%)
2017	Schultzel	10	32.1 ± 26.0	82.2 ± 15.6	123 ± 31	135 ± 17	NR
2013	Smith	26	20.9 ± 20^a^	88.3 ± 16.4	119 ± 18	159 ± 53	8 (30.8%)
2016	Smith	6	14.0 ± 10.8^a^	80.0 ± 22.9	93 ± 23	122 ± 97	4 (66.7%)

DASH: disabilities of the arm, shoulder and hand; MEPS: Mayo elbow performance score; ROM: range of motion; F/E: flexion-extension; P/S: protonation-supination.

Note: Data are reported as *n* (%) for categorical outcomes and mean ± standard deviation (SD) for continuous outcomes.

a QuickDASH score.

#### DASH and QuickDASH

The mean post-operative FullDASH score was reported in five studies, with a total of 75 patients.^3,4,6,14,16^ Scores ranged from 0 (no disability) to 100 (most severe disability). The mean post-operative FullDASH score was 25.4 (10.3) points.

The mean post-operative QuickDASH score (SD) from three studies totaling 48 patients was 15.7 (7.4) points (range 0–55)^15,17,18^ (Table 2). Similar to the FullDASH, the results can be interpreted as having no disability (score of 0) to most severe disability (score of 100). Of these studies, only Phadnis et al.^15^ classified patient outcomes based on QuickDASH scores and reported 12 patients with excellent outcomes, 4 patients with good outcomes, and no patients had fair or poor outcomes.

### ASES

The post-operative ASES score was reported in 5 (*N* = 57) studies with a range of mean scores from 61 to 72.^3,11,16–18^ Three of these studies specified the individual mean scores of the pain, functional, and patient satisfaction domains.^3,17,18^

### Pain

Post-operative pain was assessed in six studies, using a variety of tools.^1,3,6,14,17,18^ Al-Hamdani et al.^1^ used a visual analogue scale (VAS) from 0 to 10 where 0 represents a pain-free elbow.^1^ The mean VAS score was 2.6 (range 0–7), with 25% of patients being pain free, and 75% of patients experiencing some pain at a mean follow-up of 25 months.^1^ Heijink et al.^6^ used different pain categories to grade patients’ level of pain. Pain severity was graded as “none,” “mild,” “moderate,” or “severe.” At a mean of 54 months follow-up, 50% of patients reported being pain-free, 33% patients experienced mild pain, and 17% of patients experienced moderate pain.^6^ Nestorson et al.^14^ had patients assess pain as “none,” “mild,” “moderate,” or “severe” and recorded the measures in the pain domain of MEPS; 74% of patients were pain-free, 14% had mild pain, 10% had moderate level of pain, and 2% experienced severe pain at a mean follow-up of 34 months.

Smith et al.^17,18^ and Hohman et al.^3^ (*N* = 39) used the pain domain of the ASES tool. Pain levels were found to be relatively low with mean ASES pain scores of 9.9, 6, and 15, respectively.^3,17,18^ In addition to this tool, Hohman et al.^3^ also used a Likert scale, ranging from 0 (no pain) to 10 (the worst imaginable pain), although the results are not reported. Information on risk factors of pain were not reported in any of the studies pain was measured.

### Other outcome measures

Various other post-operative outcome measures were mentioned in the included studies. Oxford Elbow Score (OES) was reported in two of the included studies (*N* = 40).^1,15^ Hohman et al.^3^ reported patient satisfaction through a Likert scale, from 1 (dissatisfied) to 10 (very satisfied); the mean score was 7 out of 10. Other outcome measures included the single assessment numeric evaluation (SANE),^16^ subjective elbow value score,^18^ UCLA activity score,^18^ grip strength,^18^ EuroQol EQ5D,^17^ and the short form (SF)-36 questionnaire.^6^

### Range of motion

Post-operative range of motion (ROM) was reported in all of the included studies (*N* = 163).^1,3,4,6,11,13–18^ Seven studies (*N* = 89) reported extension deficit,^3,4,6,11,14,16,18^ nine (*N* = 131) with flexion,^3,4,6,11,14–18^ eight (*N* = 137) provided extension-flexion arc,^1,3,6,14–18^ seven (*N* = 79) reported supination,^1,3,4,6,15,16,18^ seven (*N* = 81) mentioned protonation, ^3,4,6,15–18^ and six (*N* = 85) reported supination-protonation arc.^1,3,6,15,17,18^ The ROM in the flexion and extension arc (SD) was 106° (11°), based on 9 studies with 147 patients. According to the same data, the mean ROM in the protonation and supination arc (SD) was 153° (19°) (Table 2).

#### Revision and reoperation rates

Seven revisions were reported. All seven reported revisions were conversions to total elbow arthroplasty. Only three of the included studies reported revision rate to TEA.^14,17,18^ The mean revision rate was 4% (range: 0% to 15%). Smith et al.^18^ had four patients implanted with the Sorbie-Questor prosthesis who were experiencing symptomatic loosening, revised to TEA: two for periprosthetic fractures and two for primary component loosening. Another study reported two patients who underwent revision surgery to TEA for primary prosthetic loosening. Nestorson et al.^14^ reported one patient who underwent revision due to implant loosening. The presence of a dislocation was not an indication for revision in any of the cases.

Forty-five re-operations were reported for a mean re-operation rate of 28% (range: 0% to 88%). These re-operations were comprised of the 7 aforementioned revisions and 38 other subsequent surgical procedures occurring after elbow hemiarthroplasty (Table 3). The most common reasons for re-operation included hardware irritation (mostly related to prominent olecranon hardware), stiffness, and implant loosening. The removal of olecranon hardware was performed in patients who underwent an olecranon osteotomy. Three studies decided to exclude patients that underwent revisions. For the purposes of transparency, all exclusions are *included* into the calculation of revision and re-operation rates.

**Table 3.** Associated complications with elbow hemiarthroplasty.

Complication	Number of patients	Reoperation
Stiffness	11	Yes (5)
With 60 degrees motion	1	
General stiffness	5	
Protonation/supination (mild)	5	
Ulnar attrition of coronoid process	3	
Ulnar neuropathy	13	Yes (6)
Ulnar nerve affected	7	
Persistent sensory only	3	
Persistent motor and sensory	2	
Ulnar wear	1	
Transient ulnar sensory neuropraxia	1	
Heterotopic ossification	27	Yes (4)
Implant loosening	6	Yes (3)
Type 1	4	
Type 2	1	
Type 3	1	
Primary symptomatic prosthetic loosening	2	Yes (2)^a^
Primary prosthetic loosening	2	Yes (2)^a^
Ulnar wear	12	
Mild	7	
Moderate	3	
Severe	2	
Prominent olecranon hardware	19	Yes (18)
Intraoperative olecranon fracture	2	Yes (1)
Intraoperative humeral diaphyseal fracture	1	
Undisplaced periprosthetic fractures	2	Yes (2)^a^
Subluxation of the ulnohumeral joint	1	
Calcification of the lateral collateral ligament reconstruction	1	
Radioulnar synostosis	1	
Asymptomatic radiolucency at proximal humeral stem	1	
Radiographic non-union of epicondyle	2	
Aseptic loosening	1	Yes (1)^a^
Sclerosis of ulnar sigmoid fossa	1	
Incomplete medial epicondyle union	1	
Wound necrosis	1	Yes (1)

a Revision to total elbow arthroplasty.

#### Adverse events and complications

Any event requiring surgical intervention was considered a major complication, whereas minor complications were considered adverse events. Minor AEs, such as heterotopic ossifications, radiographic olecranon wear, and transient ulnar nerve injury are treated non-invasively with pain relievers, physical therapy, and splints. The rate of AEs and complications was reported by all 11 included studies with a total of 163 patients. While the overall rate of all collective minor and major AEs was 63%, the incidence of major AEs requiring surgical intervention was 11%. Major AEs included intraoperative fractures, AEs requiring revision or re-operation (excluding hardware removal), heterotopic ossification requiring resection, or a permanent ulnar nerve injury. Of the 27 patients who experienced heterotopic ossification, 4 (15%) were symptomatic and required surgical resection. The three most common major complications were intraoperative fracture (3%), symptomatic loosening that required revision to TEA (7%), and permanent ulnar nerve injury (3%). The rate of prosthetic loosening for all patients was 3%.

The most common minor AEs were heterotopic ossifications (19%), radiographic olecranon wear (6%), and transient ulnar nerve injury (2%).

Twenty percent of total patients experienced radiographic ulnar wear at a mean of 47 months follow-up, and 7% of total patients experienced radiographic radial head wear at a mean of 36 months follow-up. None of the included studies reported if the ulnar and radial wear were symptomatic in patients. Ulnar and radial wear were not included in calculating the AEs or complications rate, as the studies did not report how many of these patients were symptomatic. Assessing wear, especially at the ulnohumeral articulation, which is predominantly affected after DHH, is often difficult due to the geometry of the ulna.^17^ Current methods based on plain X-rays are unreliable because neither the anterior–posterior (AP) or lateral radiographs can be properly standardized in terms of rotation, forearm position, and elbow extension.^7^ The included studies measured ulnar or radial wear in multiple ways, including assessing the joint space, cartilage loss, and bone loss, using lateral or anterio-posterior radiographs. Studies differed in grading the level of wear. For example, Smith et al.^17,18^ used Grade 0 (no wear) to Grade 3 (bone loss),^17,18^ whereas the metric used by Phadnis et al.^15^ consisted of none to severe (obliteration of joint space with bone erosion). The AEs and complications are summarized in Table 3.

## Discussion

This systematic review demonstrates that DHH is a reasonable option for distal humerus fractures not amenable to ORIF due to osteoporosis or extensive comminution. Moreover, DHH may be a suitable procedure for active patients, due to fewer weightbearing restrictions. This review of the literature finds that DHH results in satisfactory mean post-operative patient functional scores, along with positive mean flexion-extension and supination-protonation arcs of motion. While these positive outcomes make DHH an appealing choice, there was also a high overall complication rate of 63%. The majority of these AEs, however, were minor, asymptomatic, or had very little clinical consequences. The most common complication was heterotopic ossification (19%), which was mostly asymptomatic. The rate of major AEs was 11%. Radiographic ulnar wear occurred in 20% of patients at a mean of 47 months follow-up, and radiographic radial wear occurred in 7% of patients at a mean of 36 months follow-up. A limitation of this review is the heterogeneity and poor methodological quality of the included studies, making drawing definitive conclusions difficult.

The use of ORIF in the management of distal humeral fractures has been well documented as it often achieves stable fixation in the majority of fractures. In cases where distal humerus fractures are considered unreconstuctable, TEA is the most commonly used alternative treatment option. However, in order to improve longevity of the prosthesis, patients treated by TEA are often recommended to restrict their activities to avoid polyethylene wear and implant loosening.^19^ Generally, patients are recommended to avoid lifting with the involved upper extremity more than 5 pounds on a repetitive basis or more than 10 pounds on a single event.^20,21^ Elbow arthroplasty provides reliable outcomes, but more active patients are at risk for early mechanical failure. Two studies investigating the performance of TEA in patients younger than 40 years old both reported that 22% of patients required re-operation. One study performed re-operation within a mean of 91 months follow-up,^22^ and the other had resections done within a range of 91 months to 18 years after the initial operation.^23^ Schoch et al.^21^ reported mechanical failure in 6 of the 11 included elbows (55%). In general, TEA may be best avoided in patients under the age of 60 and may not be a good option in the more active population.^18,20^ Multiple studies have investigated the outcomes and complications of TEA for distal humeral fractures in the elderly population. One such study reported one reoperation due to a periprosthetic fracture and one case of postoperative numbness that resolved on its own.^24^ In another study, out of seven cases, there were two reported complications: one superficial wound infection and one triceps weakness.^25^ Of the 21 cases, Cobb et al.^26^ reported one revision to total elbow arthroplasty because of a fracture of the ulnar component. From the limited data available, for younger patients and patients with a more active lifestyle, DHH may offer a more suitable and appealing choice of treatment as patients are not limited by the same restrictions. However, more high-quality studies are needed to make this definitive conclusion. More specifically, the primary indication of DHH in the included studies was the presence of an acute, non-reconstructable, comminuted, intraarticular distal fracture, and/or salvage of a previous failed fixation, nonunion, or malunion. Although not the focus of this review, DHH has also been described for other indications including rheumatoid arthritis or for the treatment of tumors of the distal humerus requiring resection.^27^ Despite the wide range of indications for DHH, it is difficult to comment on the superiority or inferiority of DHH compared to TEA or ORIF due to the current lack of comparative studies.

Pain is an important consideration when comparing the clinical outcomes of DHH to TEA and ORIF. This review found that generally, patients experienced low levels or no pain after DHH. In 11 patients who underwent TEA, pain improved from 8.0 to 4.9 using the VAS.^21^ Frankle et al.^28^ compared outcomes of TEA and ORIF and reported that the average score for pain relief out of 45, as measured by the MEPS, was 40 and 43 for ORIF and TEA, respectively.

Our review reports an overall high rate of AEs and complications; however, most of the AEs are minor in nature. The rate for major complications was 11%, which comprised of intraoperative fractures, implant loosening, symptomatic heterotopic ossification requiring resection, permanent ulnar nerve injury, and any other complications requiring revision or re-operation surgery (excluding hardware removal). A systematic review and meta-analysis by Githens et al.^29^ found similar types of complication rates for ORIF and TEA. More specifically, major complications were more common after ORIF, and as such, reoperation rates were higher in the ORIF group [9%; confidence interval (CI), 4.8%–14%] as compared with TEA (6%; CI, 3.1%–8.4%), although this did not reach statistical significance. The total complication rate was higher in the TEA group because of a higher rate of minor complications. TEA resulted in 11% major and 26.6% minor complication rates, whereas ORIF resulted in 13.7% major and 20.6% minor complication rates.^29^ Formation of heterotopic bone was more common after TEA (14.7%; CI, 3.7%–25.7%) than after ORIF (4.0%; CI, 1.6%–6.4%), but was considered a minor complication unless patient returned for excision.^29^ Another study also reported a similar overall high rate of AE and complications to be 82% for TEA.^21^ Of the 11 included elbows, 6 sustained mechanical failures (5 had ulnar loosening and one had humeral loosening).^21^ Lovy et al.^30^ compared the complication rate between TEA and ORIF using information from a validated national database. The study included 33 TEA cases and 143 ORIF cases. They reported that TEA was associated with an increased odds of severe adverse events compared to ORIF (odds ratio = 1.57, *P* = 0.16), although it did not achieve statistical significance (Lovy). Infection rate was 0.7% in ORIF and 0.0% in TEA (*P* = 0.99).^30^

Besides surgical interventions such as DHH, TEA, or ORIF, distal humeral fractures in the elderly can also be managed with a conservative approach. In a study of 56 patients that were treated with cast immobilization, the mean MEPS score of the prospective and retrospective series was 83 and 86, respectively.^31^ The mean Quick-DASH scores were 34.4 and 31.3 points for the prospective and retrospective series, respectively. In total, there were six post-operative complications.^31^ Brownson et al. reported the functional outcomes and complications of conservative treatment in 44 cases.^32^ Using the Oxford elbow score (0 = worst/4 best result), the mean pain score was 2.44 (range 1–4), 2.26 (0–4) for function, and 2.04 (0–4) for psycho-social, although several patients had early dementia. Five patients underwent replacement surgery.^32^

One of the primary concerns with the DHH is articular cartilage wear of the olecranon and radial head.^15^ Ulnar and radial cartilage wear are long-term sequelae associated with DHH for the treatment of distal humeral fractures. The clinical outcomes of this type of cartilage wear are unknown, due to the lack of available high-quality data and the short follow-up period. The mean follow-up of included studies ranged from 12 months to 81 months. This short time period precludes proper assessment of the symptomatic long-term effects of cartilage wear and the revision to TEA due to this type of damage.

Another published review outlined the current technique, indications, and results of DHH.^16^ Our study is an update to this review and employs a more rigorous methodology. While Phadnis et al. provided a broad overview of DHH by detailing its historical importance, different implants used, and operative techniques, our paper provides a more focused perspective by discussing the clinical outcomes and complications of DHH for the specific indication of distal humeral fractures.

## Limitations

The poor quality of included studies and paucity of high-quality studies makes it difficult to draw definitive conclusions. The heterogeneity and lack of standardized outcome measurements and consistent follow-up are other limitations of our review. Future high-quality comparative studies comparing DHH to TEA and/or ORIF for the treatment of distal humerus fractures would be useful in determining the relative superiority of either treatment option.

## Conclusions

This review highlights DHH as a possible suitable option for distal humeral fractures, as it offers positive functional outcomes for patients with distal humeral fractures. However, the mean follow-up period of the included studies was too short to accurately assess the effect of ulnar and radial cartilage wear and need for revision to TEA.

## Supplemental Material

sj-pdf-1-sel-10.1177_17585732211023100 - Supplemental material for Outcomes and complications of distal humeral hemiarthroplasty for distal humeral fractures – A systematic review Click here for additional data file. Supplemental material, sj-pdf-1-sel-10.1177_17585732211023100 for Outcomes and complications of distal humeral hemiarthroplasty for distal humeral fractures – A systematic review by Ann M Wilfred, Shakib Akhter, Nolan S Horner, Ahmed Aljedani, Moin Khan and Bashar Alolabi in Shoulder & Elbow

sj-pdf-2-sel-10.1177_17585732211023100 - Supplemental material for Outcomes and complications of distal humeral hemiarthroplasty for distal humeral fractures – A systematic review Click here for additional data file. Supplemental material, sj-pdf-2-sel-10.1177_17585732211023100 for Outcomes and complications of distal humeral hemiarthroplasty for distal humeral fractures – A systematic review by Ann M Wilfred, Shakib Akhter, Nolan S Horner, Ahmed Aljedani, Moin Khan and Bashar Alolabi in Shoulder & Elbow

sj-pdf-3-sel-10.1177_17585732211023100 - Supplemental material for Outcomes and complications of distal humeral hemiarthroplasty for distal humeral fractures – A systematic review Click here for additional data file. Supplemental material, sj-pdf-3-sel-10.1177_17585732211023100 for Outcomes and complications of distal humeral hemiarthroplasty for distal humeral fractures – A systematic review by Ann M Wilfred, Shakib Akhter, Nolan S Horner, Ahmed Aljedani, Moin Khan and Bashar Alolabi in Shoulder & Elbow
