# Supplementary material for: Outcomes and complications of distal humeral hemiarthroplasty for distal humeral fractures – A systematic review
Source: Shoulder Elbow. 2021 Jun 17;14(1):65–74. doi: 10.1177/17585732211023100 (PMC8832700; doi:10.1177/17585732211023100)
Supplement: sj-pdf-1-sel-10.1177_17585732211023100 - Supplemental material for Outcomes and complications of distal humeral hemiarthroplasty for distal humeral fractures – A systematic review [file sj-pdf-1-sel-10.1177_17585732211023100.pdf]

Database	Search Strategy
PubMed	(((((humeral hemiarthroplasty) OR humeral arthroplasty) OR elbow hemiarthroplasty) OR elbow arthroplasty) OR elbow replacement) AND distal
EMBASE	1. exp humeral shaft/ or exp humeral head fracture/ or exp humeral supracondylar fracture/ or exp humeral head/ or exp recurrent shoulder dislocation/ or experience humeral neck/ or exp humeral condyle/ or exp distal humeral fracture/ or exp humeral neck fracture/<br>2. exp arthroplasty/<br>3. exp hemiarthoplasty/<br>4. 1 and 2<br>5. 1 and 3<br>6. Exp elbow arthroplasty/<br>7. 4 or 5 or 6<br>8. distal.mp.<br>9. 7 and 8
MEDLINE	1. exp humeral fractures/ or exp humerus/<br>2. exp arthroplasty/<br>3. exp hemiarthroplasty/<br>4. 1 and 2<br>5. 1 and 3<br>6. exp arthroplasty, replacement, elbow/<br>7. 4 or 5 or 6<br>8. distal.mp.<br>9. 7 and 8

Appendix Table 1. Search strategy for each database. All databases were searched on April 14, 2020
