# Supplementary material for: Outcomes and complications of distal humeral hemiarthroplasty for distal humeral fractures – A systematic review
Source: Shoulder Elbow. 2021 Jun 17;14(1):65–74. doi: 10.1177/17585732211023100 (PMC8832700; doi:10.1177/17585732211023100)
Supplement: sj-pdf-2-sel-10.1177_17585732211023100 - Supplemental material for Outcomes and complications of distal humeral hemiarthroplasty for distal humeral fractures – A systematic review [file sj-pdf-2-sel-10.1177_17585732211023100.pdf]

Year	First Author	Clearly stated aim	Inclusion of consecutive patients	Prospective collection of data	Endpoints appropriate to aim of study	Unbiased assessment of study endpoint	Follow-up period appropriate to study aim	Loss to follow-up less than 5%	Prospective calculation of sample size	Total score (out of 16)
2012	Adolfsson	2	1	2	0	0	2	1	0	8
2019	Al-Hamdani	0	2	0	1	0	2	1	0	6
2012	Argintar	2	2	0	0	0	2	2	0	8
2015	Heijink	1	2	0	2	0	2	1	0	8
2014	Hohman	2	2	2	2	1	2	1	0	12
2015	Nestorson	2	1	2	1	0	2	1	0	9
2005	Parsons	0	0	0	0	0	0	1	0	1
2015	Phadnis	2	2	0	1	0	2	1	0	8
2017	Schultzel	2	2	0	0	0	2	1	0	7
2013	Smith	2	2	0	1	2	2	1	0	10
2016	Smith	2	2	0	1	2	2	1	0	10

Appendix Table 2. Risk of bias assessment using the MINORS tool.
