# Supplementary material for: Outcomes and complications of distal humeral hemiarthroplasty for distal humeral fractures – A systematic review
Source: Shoulder Elbow. 2021 Jun 17;14(1):65–74. doi: 10.1177/17585732211023100 (PMC8832700; doi:10.1177/17585732211023100)
Supplement: sj-pdf-3-sel-10.1177_17585732211023100 - Supplemental material for Outcomes and complications of distal humeral hemiarthroplasty for distal humeral fractures – A systematic review [file sj-pdf-3-sel-10.1177_17585732211023100.pdf]

Year	Author	Total # of Patients Experiencing Ulnar Wear	Total # of Patients Experiencing Radial Wear	Mean follow-up
2012	Adolfsson	0	0	54
2019	Al-Hamdani	1	0	25
2012	Argintar	0	0	12
2015	Heijink	0	0	54
2014	Hohman	0	7 (Mild: 5; Moderate: 2)	36
2015	Nestorson	0	0	34.3
2005	Parsons	0	0	NR
2015	Phadnis	10 (Mild: 8 ; Moderate: 2)	3 (Mild)	35
2017	Schultzel	0	0	80
2013	Smith	13 (Grade 1: 7; Grade 2: 4; Grade 3: 2).	0	81
2016	Smith	5 (Grade 1: 2; Grade 2: 2; Grade 3: 1)	0	67

Appendix Table 3. Total number of patients experiencing radial wear or ulnar wear.
